# Altered DNA methylation is associated with aberrant gene expression in parenchymal but not airway fibroblasts isolated from individuals with COPD

**DOI:** 10.1186/s13148-018-0464-5

**Published:** 2018-03-05

**Authors:** Rachel L. Clifford, Nick Fishbane, Jamie Patel, Julia L. MacIsaac, Lisa M. McEwen, Andrew J. Fisher, Corry-Anke Brandsma, Parameswaran Nair, Michael S. Kobor, Tillie-Louise Hackett, Alan J. Knox

**Affiliations:** 1 Nottingham NIHR Biomedical Research Centre, Nottingham MRC Molecular Pathology Node, Division of Respiratory Medicine, University of Nottingham, Nottingham University Hospitals NHS Trust, City Hospital, Nottingham, UK; 2 Centre for Heart Lung Innovation, University of British Columbia, Vancouver, Canada; 3 Department of Medical Genetics, Centre for Molecular Medicine and Therapeutics, BC Children’s Hospital Research Institute, University of British Columbia, Vancouver, Canada; 4 Institute of Cellular Medicine, Newcastle University, Newcastle upon Tyne, UK; 5 Department of Pathology and Medical Biology, University of Groningen, University Medical Center, Groningen, Groningen, The Netherlands; 6 GRIAC (Groningen Research Institute of Asthma and COPD), University of Groningen, University Medical Center, Groningen, The Netherlands; 7 Firestone Institute for Respiratory Health, St Joseph’s Healthcare and Department of Medicine, McMaster University, Hamilton, Ontario Canada; 8 Department of Anaesthesiology, Pharmacology, & Therapeutics, University of British Columbia, Vancouver, Canada

**Keywords:** DNA methylation, Fibroblasts, COPD, Airway, Parenchyma

## Abstract

**Background:**

Chronic obstructive pulmonary disease (COPD) is a heterogeneous disease of the lungs that is currently the fourth leading cause of death worldwide. Genetic factors account for only a small amount of COPD risk, but epigenetic mechanisms, including DNA methylation, have the potential to mediate the interactions between an individual’s genetics and environmental exposure. DNA methylation is highly cell type-specific, and individual cell type studies of DNA methylation in COPD are sparse. Fibroblasts are present within the airway and parenchyma of the lung and contribute to the aberrant deposition of extracellular matrix in COPD. No assessment or comparison of genome-wide DNA methylation profiles in the airway and parenchymal fibroblasts from individuals with and without COPD has been undertaken. These data provide valuable insight into the molecular mechanisms contributing to COPD and the differing pathologies of small airways disease and emphysema in COPD.

**Methods:**

Genome-wide DNA methylation was evaluated at over 485,000 CpG sites using the Illumina Infinium HumanMethylation450 BeadChip array in the airway (non-COPD *n* = 8, COPD *n* = 7) and parenchymal fibroblasts (non-COPD *n* = 17, COPD *n* = 29) isolated from individuals with and without COPD. Targeted gene expression was assessed by qPCR in matched RNA samples.

**Results:**

Differentially methylated DNA regions were identified between cells isolated from individuals with and without COPD in both airway and parenchymal fibroblasts. Only in parenchymal fibroblasts was differential DNA methylation associated with differential gene expression. A second analysis of differential DNA methylation variability identified 359 individual differentially variable CpG sites in parenchymal fibroblasts. No differentially variable CpG sites were identified in the airway fibroblasts. Five differentially variable-methylated CpG sites, associated with three genes, were subsequently assessed for gene expression differences. Two genes (OAT and GRIK2) displayed significantly increased gene expression in cells isolated from individuals with COPD.

**Conclusions:**

Differential and variable DNA methylation was associated with COPD status in the parenchymal fibroblasts but not airway fibroblasts. Aberrant DNA methylation was associated with altered gene expression imparting biological function to DNA methylation changes. Changes in DNA methylation are therefore implicated in the molecular mechanisms underlying COPD pathogenesis and may represent novel therapeutic targets.

**Electronic supplementary material:**

The online version of this article (10.1186/s13148-018-0464-5) contains supplementary material, which is available to authorized users.

## Background

Chronic obstructive pulmonary disease (COPD) affects 300 million people worldwide, is currently the fourth leading cause of death in the world, and is expected to become the third leading cause of mortality worldwide by 2020 [1]. Clinically defined by airflow obstruction that is not reversible, COPD is a heterogeneous disease within the lung involving parenchymal lung destruction resulting in loss of elastic recoil (emphysema) and small airway disease [2, 3]. COPD is caused by exposure to noxious particles or gases that are predominantly introduced into the lung by cigarette smoking, or other exposures such as biomass fuels. The irreversible airflow obstruction is not reversed by cigarette smoking cessation, and to date, there are no therapies that can modify disease activity or progression. A better understanding of the molecular processes underlying the pathology of COPD is crucial to the design of novel therapeutics [4].

Genome-wide genetic association studies (GWAS) have identified four susceptibility loci for COPD that are associated with smoking behavior and have been well replicated [5–12], but each variant explains only a small amount of the risk for COPD [1]. It is therefore suggested that epigenetic processes may contribute to COPD risk by mediating the link between genetic variation and environmental exposure [1]. DNA methylation is a heritable, tissue-specific epigenetic modification to DNA that regulates gene expression [4]. It is fundamental to normal development and is known to play a crucial role in a number of chronic inflammatory diseases including cancer and aging [13]. The reversibility of DNA methylation makes it an attractive target for drug design. There is strong evidence of dysregulated DNA methylation in association with COPD [1, 13]. For example, 349 CpG sites are differentially methylated in white blood cells of individuals with COPD compared to individuals without COPD [1]. Furthermore, several studies have assessed aberrant DNA methylation in the whole lung tissue with varying levels of significance depending on sample number [13], integration with gene expression [2], and integration with GWAS results [14]. However, in terms of understanding molecular processes in COPD, these studies are limited by two factors: either they were performed in blood, having relevance as blood-based biomarkers of disease but with limited translation to lung pathology, or they were performed in the whole lung tissue where cell type-specific methylation profiles will likely have masked disease relevant alterations. Further, mixed cell populations in whole tissue complicate subsequent cell type-specific mechanistic studies to understand disease biology. An exception is the identification of 1260 CpGs differentially methylated in small airway epithelial cells between cells isolated from former smokers with and without COPD, which was associated with altered expression of 471 genes [4]. This study strongly suggests that cell type-specific alterations to DNA methylation exist in association with COPD status.

In the present study, we focused on DNA methylation in the lung fibroblasts. Fibroblasts are mesenchymal cells found in the stroma of many tissues and in the adventitia of the vasculature, airways, and parenchyma of adult lungs [15]. They are crucial for stem cell maintenance, lung repair, and the homeostasis of the extracellular matrix. The airway and parenchymal fibroblasts from COPD patients differ in physiological extracellular matrix (ECM) production [16–18], response to TGFβ [16], response to steroids [16], and proliferation rate [17]. Fibroblasts isolated from the lung parenchyma of individuals with COPD are less contractile [19, 20], less active to chemoattractant migration [19], and express and secrete increased levels of inflammatory cytokines CXCL8 and IL-6 [21] compared to parenchymal fibroblasts from individuals without COPD, suggesting they have diminished capacity to mediate repair responses potentially contributing to emphysema development and an enhanced pro-inflammatory profile. Fibroblasts isolated from the intrapulmonary airways within the lungs of COPD patients have been shown to have increased extracellular matrix deposition [16, 17]. The different pathophysiology of emphysema and small airway disease in COPD highlights the necessity to study fibroblasts from these two locations distinctly and in comparison to fully understand the molecular mechanisms underlying COPD pathogenesis.

In this study, we aimed to identify differences in genome-wide DNA methylation profiles and their associations with differences in gene expression, between the airway and parenchymal fibroblasts isolated from individuals with and without COPD, to further understand in a cell-specific manner the molecular mechanisms underlying the pathogenesis of COPD. We found that parenchymal fibroblasts, but not airway fibroblasts, had DNA methylation associated alterations in gene expression, implicating DNA methylation as a molecular mechanism underlying parenchymal dysfunction in COPD. These data provide novel evidence that the airway and parenchymal fibroblasts are epigenetically different in COPD and suggest that alterations to DNA methylation contribute to COPD pathogenesis.

## Methods

### Isolation and culture of airway and parenchymal fibroblasts

Primary cultures of airway and parenchymal fibroblasts from patients with and without COPD (airway: non-COPD *n* = 8, COPD *n* = 7; parenchymal: non-COPD *n* = 17, COPD *n* = 29, detailed demographics provided in the “Results” section) (defined by the Global Initiative for Chronic Obstructive Pulmonary Disease (GOLD) guidelines using spirometry) were established from lung biopsies or intrapulmonary airways and parenchymal lung tissue obtained from lung cancer resections (from disease-free areas) surgeries, donor lungs, and explant lungs from individuals with COPD undergoing lung transplantation. Airway and parenchymal fibroblasts were derived using the outgrowth techniques as previously described [22, 23]. Briefly, 2-mm^2^ tissue explants were placed in 6-well tissue culture plates with DMEM (Sigma) containing 10% fetal bovine serum (GIBCO, Life Technologies), penicillin (100 U/ml), streptomycin (100 μg/ml), and l-glutamine (4 mM) in a 5% CO_2_-humidified incubator. Media were replaced regularly until cellular outgrowth reached confluence. Tissue pieces were removed and destroyed, and cells were harvested using trypsin/EDTA solution (Sigma). All samples were generated from cells at passage 4 except a single airway fibroblast non-COPD donor that was collected at passage 3. Cells at the required passage were grown to confluence in 6-well plates and serum starved for 24 h prior to lysis for DNA and RNA isolation. The tissue was obtained, and cells were extracted with the approval of each of the research ethics boards for each of the academic institutions involved: Newcastle University (NRES Committee: Newcastle and North Tyneside 1 ref:11/NE/0291), McMaster University (Hamilton Integrated Research Ethics Board Ref:00–1839), University of British Columbia (Providence Health Care Research Ethics Board Ref:H13-02173), University of Nottingham (East Midlands Research Ethics Committee ref: 08/H0407/1), and University Medical Center Groningen. This study was conducted according to the national ethical and professional guidelines on the use human body material (“Code of conduct; Dutch federation of biomedical scientific societies”; https://www.federa.org/codes-conduct) and the Research Code of the University Medical Center Groningen (https://www.umcg.nl/EN/Research/Researchers/General/ResearchCode/Paginas/default.aspx). The demographics of the subject lung fibroblasts assessed are given in Table 1.

**Table 1:** Donor demographics

	Airway fibroblasts			Parenchymal fibroblasts			
	Non-COPD	COPD	Non-COPD vs COPD *p* value	Non-COPD	COPD	Non-COPD vs COPD *p* value	Airway vs parenchymal COPD *p* value
*N*	8	7		17	29		
Gender (M/F)	2/6	6/1	0.0187	9/8	18/11	0.544	0.2336
Age Mean (SD)	63.36 (10.06)	68 (5.63)	0.3097	65.06 (11.31)	65.1 (9.60)	0.9994	0.4489
Pack-years Mean (SD)	21.6 (18.89)	53.4 (29.84)	0.0221	31.8 (25.52)	39.8 (19.94)	0.3345	0.1798
FEV1% Mean (SD)	100.8 (11)	69.7 (18.5)	0.0046	95.6 (17.2)	47.8 (26.3)	< 0.0001	0.0457
FEV1/FVC % Mean (SD)	77.42 (7.2)	59.8 (12)	0.0035	76.6 (6.4)	44.8 (15.5)	< 0.0001	0.0182
GOLD stage	NA	2× GS1, 3× GS2, 2× GS3	NA	NA	3× GS1, 10× GS2, 3× GS3, 13× GS4	NA	NA

*M* male, *F* female, *COPD* chronic obstructive pulmonary disease, *SD* standard deviation, *FEV* forced expiratory volume, *FVC* forced vital capacity, *NA* not applicable

### DNA and RNA isolation

DNA and RNA were simultaneously isolated from each sample using the AllPrep DNA/RNA Mini Kit (Qiagen) as per manufacturer’s instructions and assessed for quality and quantity using a NanoDrop™ 8000 Spectrophotometer (Thermo Fisher Scientific).

### Bisulfite conversion and DNA methylation arrays

Seven hundred fifty nanograms of purified genomic DNA was bisulfite converted using the EZ DNA Methylation Kit (Zymo Research) as per the manufacturer’s instructions. Specific incubation conditions for the Illumina Infinium Methylation Assay were used as per the manufacturer’s protocol Appendix. Samples were eluted in 12 μl. Bisulfite-converted DNA was assessed for concentration and quality using a NanoDrop™ 8000 Spectrophotometer (Thermo Fisher Scientific), and 160 ng of the conversion product was used for genome-wide DNA methylation quantification at over 485,000 CpG sites using the Illumina Infinium HumanMethylation450 BeadChip array, according to the manufacturer’s protocols.

### Data quality control and normalization

IDAT files produced by GenomeStudio were imported into the R statistical software (version 3.2.1) using the minfi package (v. 1.14.0) [24]. The 65 known quality control SNP probes were used to cluster all samples to detect anomalies within the samples from the same donor. Probes were excluded from further analysis according to several criteria: first, 1402 probes were found to have either a detection *p* value < 0.05 in at least 1% of samples or had less than three bead count in at least 5% of samples; second, the 65 SNP probes; third, 59,593 probes were found to be cross-hybridized to other parts of the genome, known to be polymorphic at the CpG or examine single nucleotide polymorphisms [25]; finally, 9925 sites on the X chromosome or the Y chromosome. Four hundred fourteen thousand five hundred ninety-two probes remained for analysis.

Filtered probes were normalized using the funtooNorm algorithm [26], which extends the funNorm procedure [27] and is purported to correct for unwanted variation while preserving important differences in methylation patterns between different cell types. We employed the normalization option of principal components regression with five principal components. Two values of DNA methylation were used, beta values (*β* values) and *M* values. *β* values are the ratio of all methylated probe intensities over total signal intensities (methylated and unmethylated) and have a range from 0 to 1. They approximately represent percent methylation. *M* values are the log transformation of *β* values and are more statistically robust [28]. All statistical analyses were performed using *M* values, while *β* values were used for visualization and interpretability purposes. Principal component analysis was performed for quality control of the *M* values.

### Differential DNA methylation analysis

All statistical analyses were performed using R statistical software (version 3.2.1). Probes with DNA methylation levels significantly different between non-COPD donors and COPD donors in the airway and parenchymal fibroblasts separately were identified using the limma package using multivariable linear regression on *M* values adjusting for covariates [29] followed by control of the *p* values of the main effect coefficient for false discovery rate via the Benjamini-Hochberg procedure [30] across all CpG sites on the array. For airway fibroblasts, biological sex and smoking pack-years were included as covariates. As no independently significant sites were identified using limma, we looked at aggregated sites to identify differentially methylated regions (DMRs) using the DMRcate package in R [31], which uses Gaussian kernel smoothing to find patterns of differential methylation, agnostic to genomic annotation. We used the authors’ recommended bandwidth (*λ*) of 1000 base pairs and scaling factor (*C*) of 2. For airway fibroblasts, we used a nominal *p* value cutoff of *p* < 0.005, which yielded an analysis of 3432 CpG sites. For the parenchymal fibroblast analysis, all donors were sex- and pack-years matched so a more lenient nominal *p* value cutoff of *p* < 0.01 was used, which yielded an analysis of 2837 CpG sites.

### Variable DNA methylation analysis

Variable DNA methylation analysis was performed using the iEVORA algorithm in R [32] and the recommended *q* value cutoff for variably methylated points (DVPs) of *p* < 0.001.

### Reverse transcription and qPCR

0.5 μg of RNA was reverse transcribed using SuperScript IV (Invitrogen), as per the manufacturer’s instructions. The resulting 20 μl cDNA samples were diluted to a total volume of 200 μl using nuclease-free water. cDNA was amplified using PerfeCTa SYBR Green FastMix (Quanta bio), with 2 μl template and 200 nM primers in a 10-μl reaction using a Stratagene Mx3000P/3005P system. Thermal cycler conditions included incubation at 95 °C for 10 min, followed by 40 cycles of 95 °C for 10 s, 60 °C for 30 s, and 72 °C for 20 s. Data were collected in MxPro; a single product was confirmed by melt curve analysis, and Ct values were exported to Excel for analysis. Expression was expressed by the ΔΔCt method relative to β_2_-microglobulin (β_2_M) Ct and mean non-COPD target/β_2_M ΔCt. Each cDNA was run in triplicate for both the target gene and housekeeping gene. The mean of the triplicate Cts were taken and the ΔCt between target and housekeeping gene calculated. The mean ΔCt for non-COPD samples was calculated and ΔΔCts were calculated relative to the mean non-COPD ΔCt. Primer sequences are as follows: β_2_-microglobulin, forward 5′-AATCCAAATGCGGCATCT-3′, reverse 5′-GAGTATGCCTGCCGTGTG-3′; TMEM44, forward 5′-GGCACTGGACCTCGCTATTA-3′, reverse 5′-CAGGCTCGATGGTCAGCTC-3′; NXN, forward 5′-AGACTCTGTTTGGGAGCACG-3′, reverse 5′-TGACTTTGCGAAAGCCATGC-3′; HLX, forward 5′-CGTTTCCAGGTCCCTATGCT-3′, reverse 5′-CGGTTCTGGAACCACACCTT-3′; SPON2, forward 5′-TCCCACGTGGTTGCAGATAC-3′, reverse 5′-TTCCGAAACCGCCCCATTTA-3′; TRPV3, forward 5′-GTGGCCTGCCTGGCG-3′, reverse 5′-GCTTTCATGGCTGGTGAGGT-3′; OAT, forward 5′-CGCTGTCAGATCTGTGGTTT-3′, reverse 5′-ACTCCGCGACTAAGTACA-3′; and GRIK2, forward 5′-CATGCAGCAAGGTTCTGAGC-3′, reverse 5′-CACTGTCAGAAAGGCGGCTA.

### Bisulfite PCR-pyrosequencing

Bisulfite PCR-pyrosequencing was used to validate differences in DNA methylation cg16009558 (GRIK2). Bisulfite PCR-pyrosequencing assays were designed with PyroMark Assay Design 2.0 (Qiagen). The regions of interest were amplified by PCR using the HotstarTaq DNA polymerase kit (Qiagen) as follows: 15 min at 95 °C (to activate the Taq polymerase), 45 cycles of 95 °C for 30s, 58 °C for 30s, and 72 °C for 30s, and a 5 min 72 °C extension step. For pyrosequencing, single-stranded DNA was prepared from the PCR product with the Pyromark™ Vacuum Prep Workstation (Qiagen), and sequencing was performed using sequencing primers on a Pyromark™ Q24 pyrosequencer (Qiagen). The quantitative levels of methylation for each CpG dinucleotide were calculated with Pyromark Q24 software (Qiagen). Primer sequences were forward biotinylated-5′-ATTTTAGTTTTTTTTATTTAATTTTGGTTT-3′, reverse 5′-CAAAAATTTTACCAAACCCTATTCTACT-3′, sequencing 5′-ACACTACTACACAACTTCTAA-3′. Samples were the same bisulfite converted samples used on the array. COPD versus non-COPD donors were demographically matched.

## Results

### Demographic data

Genome-wide DNA methylation data analysis was performed using airway fibroblasts from 7 patients with COPD and 8 non-COPD controls and parenchymal fibroblasts from 29 patients with COPD and 17 non-COPD controls (Table 1). Due to the nature of the surgical procedures from which the lung tissue was taken, there were no paired airway and parenchymal cells from the same donor in the current dataset. There were no significant differences between COPD and non-COPD parenchymal fibroblast samples based on their age, sex, or pack-years smoking history. There were significant differences between COPD and non-COPD airway fibroblast samples based on their sex and pack-years smoking history, and these were included as covariates in the statistical models. There was also a significant difference in COPD severity between the airway and parenchymal fibroblasts as defined by FEV1% and FEV1/FVC%.

### Differentially methylated DNA regions were associated with COPD status in the airway and parenchymal fibroblasts

To understand whether differential DNA methylation was associated with COPD in either airway or parenchymal fibroblasts, we assessed genomic DNA methylation using the Illumina Infinium HumanMethylation450 BeadChip. After quality control, 414,592 probes remained for analysis.

**Fig. 1:**
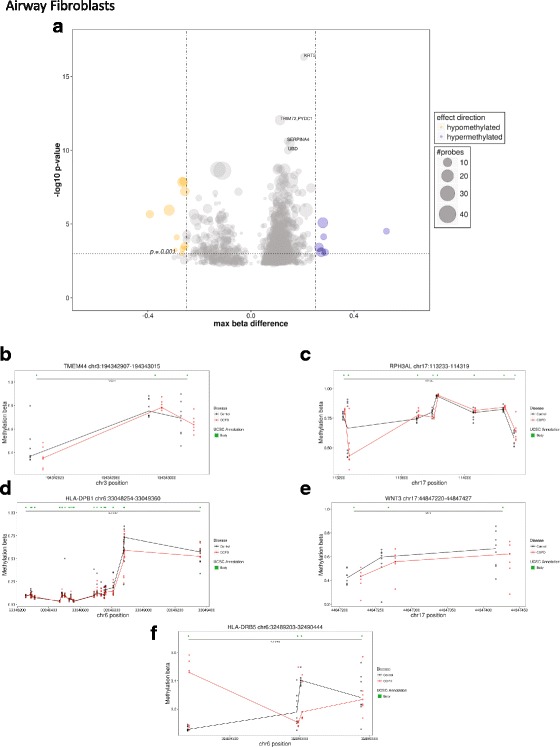
Regional DNA methylation differs in airway fibroblasts isolated from donors with COPD versus non-COPD. **a** Summary of regional DNA methylation differences between DNA isolated from donors with and without COPD. Circle size represents the number of probes per regions. Orange = hypomethylated in COPD samples, blue = hypermethylated in COPD. **b**–**f** Detailed plots of the five regions with the greatest maximum difference in DNA methylation between DNA isolated from donors with and without COPD

We looked to identify individually differentially methylated CpG sites with linear regression, with COPD status as the variable of interest. Linear modeling did not identify any independently significant sites, after adjusting for multiple tests, in either airway or parenchymal fibroblasts. Subsequently, we assessed differential methylation on aggregated sites using the regional DNA methylation R package DMRcate [31], including all CpGs specified by a nominal *p* value limit of 0.005 for airway fibroblasts (3432 CpG sites) and a more lenient 0.01 for parenchymal fibroblasts (2837 CpG sites). In airway fibroblasts, 887 differentially methylated regions were identified in COPD that contained at least three CpG sites. Six hundred fifty-two of these regions were annotated to a known gene, and 35 had a maximum difference in DNA methylation (i.e., at least one probe displayed a mean difference in methylation) of 20% (Δ*β* = 0.2) (Fig. 1a and Additional file 1: Table S1). Twelve of these regions had increased DNA methylation associated with COPD, while in 23 regions, DNA methylation was decreased with COPD status. The five regions with the largest maximum difference in DNA methylation were associated with the genes: *TMEM44* (Fig. 1b, max Δ*β* = 0.26),* RPH3AL* (Fig. 1c, max Δ*β* = 0.39), *HLA-DP1* (Fig. 1d, max Δ*β* = 0.31), *WNT3A* (Fig. 1e, max Δ*β* = 0.27), and *HLA-DRB5* (Fig. 1f, max Δ*β* = 0.28). The regions associated with *TMEM44*, *RPH3AL*, and *HLA-DRB5* did not display a consistent hypomethylation or hypermethylation association with COPD across all of the region CpGs. However, the regions associated with *HLA-DPB1* and *WNT3* displayed consistent hypomethylation in association with COPD status.

**Fig. 2:**
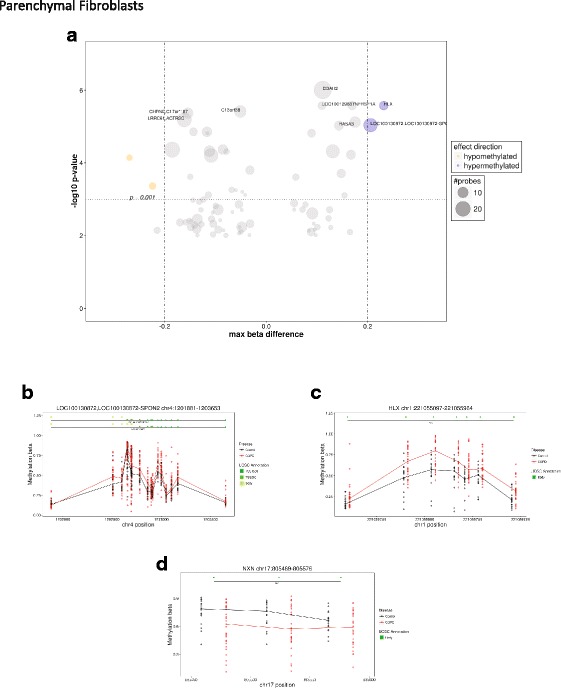
Regional DNA methylation differs in parenchymal fibroblasts isolated from donors with COPD versus non-COPD. **a** Summary of regional DNA methylation differences between DNA isolated from donors with and without COPD. Circle size represents the number of probes per regions. Orange = hypomethylated in COPD samples, blue = hypermethylated in COPD. **b**–**d** Detailed plots of the three regions with a maximum difference in DNA methylation between DNA isolated from donors with and without COPD of greater than 20% (beta value difference of 0.2)

In parenchymal fibroblasts, we identified 44 DNA differentially methylated regions containing at least three CpG sites, 39 of which were annotated to a gene but only three with a maximum Δ*β* of 0.2 (Fig. 2a and Additional file 2: Table S2). Two regions associated with genes *HLX* (Fig. 2b) and *LOC100130872-SPON2* (Fig. 2c) were hypermethylated with COPD status while a single region associated with *NXN* was hypomethylated (Fig. 2d).

### Differentially methylated DNA regions were associated with changes in steady-state gene expression with COPD status in parenchymal but not airway fibroblasts

**Fig. 3:**
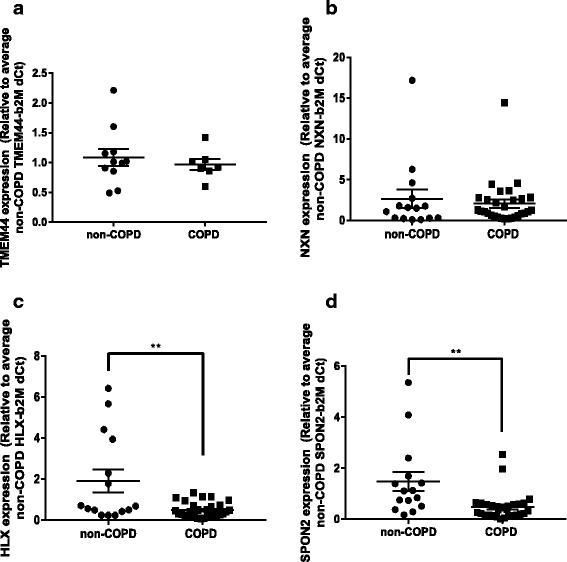
Expression of genes associated with differentially methylated DNA regions. **a** *TMEM44* expression in airway fibroblasts isolated from individuals with and without COPD. **b** *NXN* expression in parenchymal fibroblasts isolated from individuals with and without COPD. **c** *HLX* expression in parenchymal fibroblasts isolated from individuals with and without COPD. **d** *SPON2* expression in parenchymal fibroblasts isolated from individuals with and without COPD. **p < 0.01 compared with non-COPD control by unpaired *t* test, *n* = 15 non-COPD, *n* = 29 COPD

To infer a biological significance of the differentially methylated DNA regions on cell function in COPD, we performed targeted gene expression analysis for the five airway fibroblast regions with the largest maximum difference in DNA methylation and the three parenchymal fibroblasts regions. In airway fibroblasts, gene expression of *RPH3AL*, *HLA-DP1*, *WNT3A*, and *HLADRB5* was not detectable. Primer functionality was confirmed by positive amplification in cDNA from the whole lung samples (data not shown). *TMEM44* expression was detected but showed no difference in expression between cells isolated from individuals with COPD versus those without COPD (Fig. 3a).

In parenchymal fibroblasts, all transcripts except *LOC100130872* were detectable. *NXN* showed no difference in expression between cells isolated from individuals with COPD versus those without COPD (Fig. 3b). However, both *HLX* (Fig. 3c, *p* value = 0.0011 unpaired *t* test) and *SPON2* (Fig. 3d, *p* value = 0.0016 unpaired *t* test) showed a significant decrease in gene expression in cells isolated from individuals with COPD versus those without COPD.

### Variable DNA methylation is more strongly associated with differential steady-state gene expression with COPD status in parenchymal fibroblasts

Identifying differentially methylated CpG sites refers to comparing mean DNA methylation between cases and controls and is a standard analytical approach for identifying disease-associated CpG sites. Recently, the importance of increased DNA methylation variability has been identified as a predictor of progression to neoplasia in precursor cervical cancer legions [32] and has been associated with type 1 diabetes in three immune effector cell types [33]. Differentially variable CpG positions (DVPs) can identify larger differences in CpG methylation although in a smaller number of the samples [33].

**Fig. 4:**
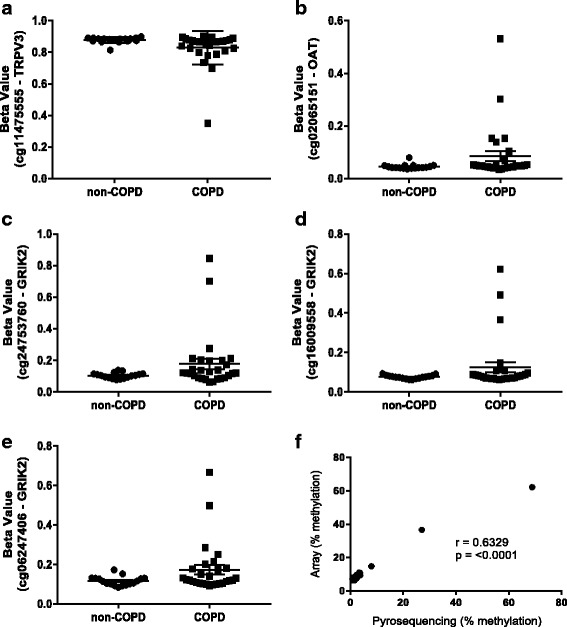
CpG methylation is differentially variable in parenchymal fibroblasts isolated from donors with COPD versus non-COPD. DNA methylation array data for five CpGs identified as differentially variable in association with COPD status. **a** cg11475555 within 200 bp of the *TRPV3* gene transcription start site. **b** cg02065151 within 200 bp of the *OAT* gene transcription start site. **c** cg24753760. **d** cg16009558. **e** cg06247406 all within 200 bp of the *GRIK2* gene transcription start site. **f** Pyrosequencing validation of cg16009559

As such, we assessed DNA methylation variability using the iEVORA algorithm in R and the association with COPD status in the airway and parenchymal fibroblasts. Using the recommended *p* value cutoff of 0.001, no DVPs were identified in airway fibroblasts; however, 359 CpG sites were identified in parenchymal fibroblasts. Three hundred twenty-seven of these CpG sites displayed greater variability (as assessed by a larger standard deviation) in cell samples isolated from individuals with COPD, 32 CpGs displayed greater variability in non-COPD cells (Additional file 3: Figure S1a). The majority of DVPs were located within CpG islands, but the distribution of location did not differ from the distribution of the full analysis probes set (Additional file 3: Figure S1b). Two hundred eighty-seven DVPs were gene annotated, with 45 within 200 bp of a transcription start site. We hypothesized that if the variation in CpG methylation is contributing to dysfunctional gene expression in parenchymal fibroblasts in COPD, the CpG sites would have relatively stable methylation (low variation) in cells isolated from individuals without COPD and become more variable in cells isolated from individuals with COPD. We therefore followed these criteria to identify CpG sites most likely to be associated with dysfunctional gene expression: (1) CpGs with greater variation in cells isolated from individuals with COPD than those without, *n* = 327; (2) CpGs with stable methylation in cells from individuals without COPD as represented by a standard deviation across samples of < 0.03, *n* = 261; (3) CpGs with the greatest variability in cells isolated from individuals with COPD, as represented by a standard deviation > 0.1, *n* = 40; and (4) CpGs are within 200 bp of a transcription start site and potentially most likely to modulate gene expression, *n* = 5. These remaining five CpG sites were annotated to the promoters of three separate genes, *TRPV3 *(cg11475555, Fig. 4a), *OAT* (cg02065151, Fig. 4b), *GRIK2* (cg24753760, Fig. 4c; cg16009558, Fig. 4d; and cg06247406, Fig. 4e). *GRIK2* CpG cg16009558 was validated by pyrosequencing and confirmed a significant correlation between pyrosequencing and array methylation (*r* = 0.6329, *p* value = < 0.001, Fig. 4f).

**Fig. 5:**
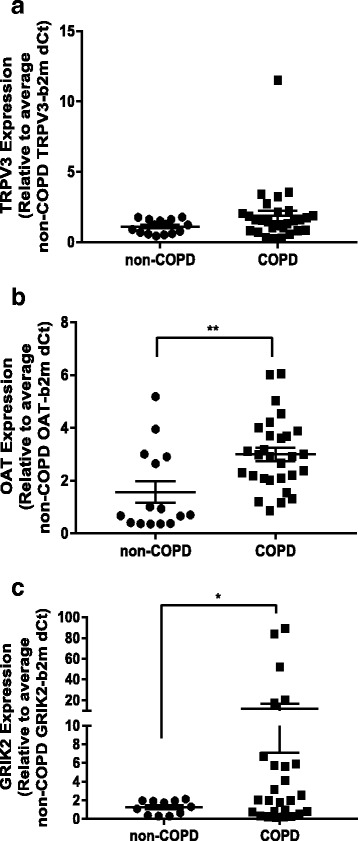
Variable CpG methylation in parenchymal fibroblasts from COPD donors is associated with differential gene expression. Gene expression data generated by qPCR for the three genes associated with variable CpG site methylation in parenchymal fibroblasts from COPD donors. **a** *TRPV3*. **b** *OAT*. **c** *GRIK2*. *p < 0.05, **p < 0.01 compared with non-COPD control by Welch’s *t* test, *n* = 15 non-COPD, *n* = 29 COPD

**Fig. 6:**
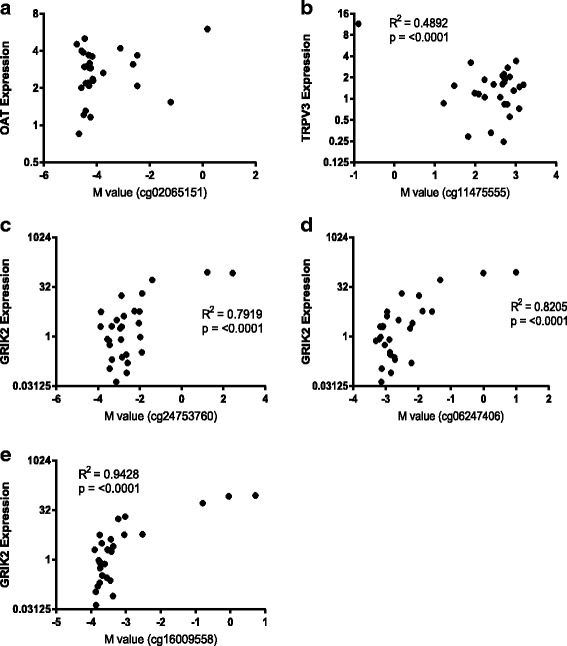
*GRIK2* CpG site methylation correlates with gene expression. Correlations between gene expression levels and DNA methylation (here represented as *M* values, the log transformation of *β* values which are more statistically robust) for the three differentially variable CpG sites/genes. **a** *OAT.* **b** *TRPV3*. **c**–**e** *GRIK2*. *R*^2^ values and *p* values are shown within the plots

We assessed the potential biological relevance of variable DNA methylation at these sites by measuring steady-state gene expression. While *TRPV3* showed a non-significant trend toward increased gene expression in cells isolated from individuals with COPD (*p* = 0.0618 Welch’s *t* test, Fig. 5a), both *OAT* (*p* = 0.0109 Welch’s *t* test, Fig. 5b) and *GRIK2* (*p* = 0.0358 Welch’s *t* test, Fig. 5c) showed a significant increase in gene expression in cells isolated from individuals with COPD versus those without COPD. In particular, in cells from four COPD donors, *GRIK2* showed a greater than 20-fold increase in gene expression. Subsequently, we asked, for these specific genes, whether expression correlated with DNA methylation in parenchymal fibroblasts isolated from individuals with COPD. *OAT* expression did not correlate with methylation of cg02065151 (Fig. 6a). Although *TRPV3* showed a non-significant trend toward increased gene expression between parenchymal fibroblasts isolated from individuals with and without COPD, *TRPV3* expression did correlate with cg11475555 methylation, suggesting DNA methylation may play a role in regulating *TRPV3 *expression (Fig. 6b). The expression of *GRIK2* strongly correlated with methylation of all three *GRIK2* CpGs determined as DVPs in COPD (cg24753760, Fig. 6c; cg06247406, Fig. 6d; cg16009558, Fig. 6e), highly suggestive of aberrant *GRIK2* CpG methylation leading to increased *GRIK2* expression in COPD parenchymal fibroblasts.

## Discussion

The main novel findings of this study are that alterations to individual CpG site methylation occur with COPD status in lung cells, that dysfunction is more strongly associated with cell function (gene expression) in parenchymal fibroblasts than airway fibroblasts and that variability in DNA methylation may represent a stronger analytical method to identify aberrant DNA methylation associated gene expression than differential methylation in a heterogeneous disease like COPD.

DNA methylation is considered to be an important biological mechanism, which integrates genetic and environmental risk factors contributing to COPD pathogenesis. DNA methylation is aberrant in the blood and whole lung tissue DNA isolated from individuals with COPD; however, these mixed cell samples, and with regard to blood separation from the site of disease, complicate defining molecular mechanisms underlying COPD pathogenesis. Fibroblasts are critical to the deposition of the physiological extracellular matrix (ECM) and are considered to contribute to pathological ECM scar tissue deposition in many obstructive airway diseases including COPD. Fibroblasts reside within the lung parenchyma and airways, and it is becoming increasingly clear that these two locations define a distinct phenotype of fibroblast that likely contributes differentially to lung disease pathogenesis. In this paper, we use purified populations of the airway and parenchymal fibroblasts from individuals with and without COPD to identify disease-associated, cell type-specific alterations to DNA methylation.

We have considered whether any technical issues may have affected our results. Our study was performed on human airway and parenchymal fibroblasts, expanded in vitro to passage 4 (except a single airway non-COPD donor that was collected at passage 3). This provides the benefit of a single cell type population for DNA methylation analysis, which we know to be highly sensitive to mixed cell populations. By using cells at the same passage, grown under the same conditions, it is unlikely that the differences in DNA methylation and gene expression are due to cell culture effects. Furthermore, the cells used in the study were collected from four different sites to minimize isolation and culture technique effects. Secondly performing the Illumina Infinium HumanMethylation450 BeadChip array on bisulfite DNA does not allow 5-methylcytosine to be distinguished from 5-hydroxymethylcytosine. 5-Hydroxymethylcytosine is an oxidized version of 5-methylcytosine produced by Ten-eleven translocation (TET) enzymes as an intermediate base during DNA demethylation. 5-Hydroxymethylcytosine is associated with gene transcription and gene translation but via potentially different mechanisms to 5-methylcytosine. Further investigation of the mechanisms by which DNA methylation regulates gene expression in the targets identified here is warranted and should include Tet-assisted bisulfite pyrosequencing to distinguish between 5-methylcytosine and 5-hydroxymethylcytosine. Finally, the Illumina Infinium HumanMethylation450 BeadChip array, while covering a valid representation of the genome, is not truly genome-wide, and an analysis using bisulfite sequencing or the more recently released Illumina EPIC array may highlight further modifications to fibroblast DNA methylation in association with COPD status.

Our initial linear modeling approaches did not identify any individual differentially methylated CpG sites associated with COPD, potentially due to the large number of features and low sample number, despite the current study being the largest to date of purified cell types in COPD. There are data reduction techniques that we could have used, for example, selecting CpG sites within close (200/1500 bp) proximity to transcription start sites, as these are considered more likely to drive transcriptional changes. However, a secondary part of this study was to also understand how different types of DNA methylation, for example, promoter versus gene body, affected steady-state gene expression. For this reason, we chose to undertake regional analysis using the R package DMRcate, considering that while individual site differences may be small, if they are persistent across a region, the power to detect them will be greater. The regional analysis did identify regions of DNA differentially methylated between cells isolated from individuals with and without COPD. Furthermore, the location of these regions differed between airway and parenchymal fibroblasts, confirming that different molecular mechanisms are induced in these two cell types in association with COPD.

To assess the likelihood of statistically significant differentially methylated DNA regions having downstream biological effects, the absolute difference between the means of the *β* values of the COPD and non-COPD were calculated for each CpG within a region and referred to as the delta beta (Δ*β*). We considered a maximum difference in methylation of greater than 20%, that is, at least one CpG site within the designated region must have had a mean difference in methylation between samples from COPD and non-COPD donors of greater than 20% (Δ*β* = 0.2), to be of biological interest. After this filtering step, 45 regions remained of interest in the airway fibroblasts, while only 3 remained for parenchymal fibroblasts. This suggested that airway fibroblasts are likely predisposed to larger alterations in DNA methylation than parenchymal fibroblasts and therefore that they may contribute to a greater extent to COPD pathogenesis than previously considered. However, to understand the biological significance of these DNA methylation differences in greater detail, it was important to establish whether DNA methylation changes were altering gene expression. We assessed gene expression for all three parenchymal fibroblast regions and the five regions from airway fibroblasts with the largest maximum difference in DNA methylation between non-COPD and COPD samples. None of the airway fibroblast regions was associated with a difference in steady-state gene expression, while two of the three parenchymal regions did coincide with a significant difference in gene expression. This highlights the necessity for integrating DNA methylation and gene expression data to confer biological importance. However, it does not exclude that the differences in DNA methylation in the airway fibroblasts may affect gene expression at regions we did not assess by qPCR or in response to, for example, lung/cell exposure to environmental COPD risk factors (cigarette smoke/air pollution), and this warrants further investigation. Of interest, although one of the significant parenchymal fibroblast regions was within 1500 bp of the transcription start site for *SPON2*, the region for *HLX*, which also showed a significant difference in gene expression, is located within the gene body, emphasizing that location within the gene also does not necessarily confer effect at the gene expression level. We isolated DNA and RNA simultaneously from the same samples, a valuable resource, and a limitation of this study is that we analyzed our RNA by targeted qPCR rather than genome-wide microarray or sequencing, which would have been interesting to gain more information regarding the association between gene expression and DNA methylation.

Evidence for a greater dysfunction in DNA methylation regulated gene expression in parenchymal rather than airway fibroblasts was further strengthened by a secondary analysis of DNA methylation variability that has previously been validated in other diseases [32, 33]. Identifying differentially methylated CpG sites compares the mean level of DNA methylation between cases and control while assessment of differential variability (DVPs) essentially identifies individual sites displaying “epigenetic outliers” in heterogeneous populations. The current analysis did not identify any DVPs in airway fibroblasts but did identify 359 DVPs in parenchymal fibroblasts. As genome-wide gene expression was not available for these samples to allow full integration to DNA methylation data, we used a filtering process to identify those CpG sites we hypothesized to be the most likely to identify differential disease-associated gene expression (gene annotated, greater variability in COPD than non-COPD, stable variation in non-COPD and largest variation in COPD, within 200 bp of a transcription start site) and assessed the expression of the identified genes by qPCR. The five sites that remained associated with three different genes, two of which showed a significant difference in expression between samples from COPD and non-COPD donors (*OAT* and *GRIK2*) and a third that displayed a strong trend (*p* = 0.0618) toward differential expression (*TRPV3*). The higher success rate of identifying differences in DNA methylation associated with differential gene expression by variation analysis than absolute differential analysis suggests that differential variation analysis may be a preferable method for identifying DNA methylation regulated alterations in gene expression associated with heterogeneous diseases like COPD. However, we acknowledge that a full integration of genome-wide DNA methylation and gene expression data and permutation testing is required to definitively prove this finding.

The largest difference in gene expression was seen for *GRIK2* with some donors displaying a greater than 80-fold increase in expression compared to non-COPD donors. *GRIK2* expression also directly correlated with DNA methylation levels in matched donor samples, indicating that DNA methylation is tightly associated with *GRIK2* gene expression. The direct relationship between both *GRIK2* and *OAT* expression and DNA methylation was surprising, that is, for both *OAT* and *GRIK2*, DNA methylation was higher in samples from COPD donors than non-COPD donors, a phenomenon at gene promoters that is normally associated with gene repression. Despite this, qPCR data showed increased expression of both *OAT* and *GRIK2* in samples from COPD donors. Again, this highlights that although general trends have been identified for the regulation of gene expression by DNA methylation, the more studies that are performed, the more exceptions to those trends arise and the more important it is to look at DNA methylation in context with gene expression data rather than as an indicator of gene expression. Furthermore, future studies are warranted to understand the mechanisms that mediate the relationship between DNA methylation and gene expression. In addition to differences in 5-methylcytosine and 5-hydroxymethylcytosine ratios mentioned above, these could include associations with methyl-DNA binding proteins, modifications to histone tails, and the ability to recruit specific transcription factors. This is likely to not only be DNA region specific but also cell type and disease context specific, and regimens to include these mechanistic studies should be included in future studies of DNA methylation in COPD.

*GRIK2* belongs to the kainate family of glutamate receptors, and a small nucleotide polymorphism (rs6570989) in *GRIK2* has been associated with a status of “current smoker” [34]. Our parenchymal fibroblast cohort was matched for smoking pack-years but did include a mixture of current, ex-, and non-smokers, and we were therefore interested in whether the variability in DNA methylation was driven by smoking history. However, neither *GRIK2* DNA methylation levels nor gene expression levels correlated with pack-years (Additional file 4: Figure S2a and b) or smoking status (Additional file 4: Figure S2c and d). We also assessed *GRIK2* single nucleotide polymorphisms (SNPs) in the donors and the T allele SNP associated with current smoking was only identified in a single non-COPD donor, together suggesting that COPD status rather smoking status and genotype are driving the observed association between *GRIK2* DNA methylation and gene expression.

## Conclusions

To conclude, we show that differences in DNA methylation are associated with COPD status and that these alterations can be associated with differences in functional gene expression for specific genes. Differences in DNA methylation profiles differ between airway and parenchymal fibroblasts suggesting that DNA methylation contributes differently to disease pathology in these two distinct tissue types with different functions within the lung. The association between gene expression and DNA methylation was complex and not predictable by the traditional dogma of DNA methylation regulated gene expression. Finally, assessing differentially variable DNA methylation may be a better approach to identifying aberrant DNA methylation regulated gene expression in heterogeneous diseases such as COPD.

## Additional files

Additional file 1: **Table S1.** (.MWD): DNA regions differentially methylated with COPD status in airway fibroblasts. Summary of the 35 gene annotated regions containing a minimum of three CpG probes and a maximum difference in DNA methylation of at least 20% (difference in beta value of 0.2). A positive delta beta identifies decreased DNA methylation in cells isolated from individuals with COPD, while a negative value identifies increased DNA methylation in cells isolated from individuals with COPD. (DOCX 18 kb)

Additional file 2: **Table S2.** (.MWD): DNA regions differentially methylated with COPD status in parenchymal fibroblasts. Summary of the three gene annotated regions containing a minimum of three CpG probes and a maximum difference in DNA methylation of at least 20% (difference in beta value of 0.2). A positive delta beta identifies decreased DNA methylation in cells isolated from individuals with COPD, while a negative value identifies increased DNA methylation in cells isolated from individuals with COPD. (DOCX 14 kb)

Additional file 3: **Figure S1.** (.EPS): CpG methylation variation in parenchymal fibroblasts in COPD samples. A) Plot of the difference in methylation standard deviation of samples from donors with COPD and donors without COPD at the 359 CpGs differentially variably methylated between samples from donors with COPD and donors without COPD. B) Representation of the CpG type with which DVPs were associated versus CpG types of all CpGs included in the analysis. (EPS 1922 kb)

Additional file 4: **Figure S2.** (.EPS): GRIK2 methylation and expression do not correlate with smoking pack-years or smoking status. A) Correlation plot of GRIK2 cg16006558 methylation with smoking pack years. B) Correlation plot of GRIK2 expression with smoking pack-years. Neither CpG methylation nor gene expression correlates with smoking pack-years. C) GRIK2 expression separated by smoking status. D) GRIK2 CpG methylation separated by smoking status. Smoking status does not drive alterations to GRIK2 DNA methylation or gene expression. (EPS 2269 kb)

## Abbreviations

COPD: Chronic obstructive pulmonary disease
DVP: Variably methylated points
ECM: Extracellular matrix
FEV: Forced expiratory volume
FVC: forced vital capacity
GOLD: Global Initiative for Chronic Obstructive Pulmonary Disease
GWAS: Genome-wide genetic association studies
SD: Standard deviation
TGFβ: Transforming growth factor beta
Δβ: Delta beta
