# Supplementary material for: Altered DNA methylation is associated with aberrant gene expression in parenchymal but not airway fibroblasts isolated from individuals with COPD
Source: Clin Epigenetics. 2018 Mar 5;10:32. doi: 10.1186/s13148-018-0464-5 (PMC5838860; doi:10.1186/s13148-018-0464-5)
Supplement: Supplementary file 1 — Table S1. (.MWD): DNA regions differentially methylated with COPD status in airway fibroblasts. Summary of the 35 gene annotated regions containing a minimum of three CpG probes and a maximum difference in DNA methylation of at least 20% (difference in beta value of 0.2). A positive delta beta identifies decreased DNA methylation in cells isolated from individuals with COPD, while a negative value identifies increased DNA methylation in cells isolated from individuals with COPD. (DOCX 18 kb) [file 13148_2018_464_MOESM1_ESM.docx]

| Gene Associated | Gene region  location | hg19 coordinate | Number of probes | Minimum p value | Mean p value | Maximum beta value difference |
| --- | --- | --- | --- | --- | --- | --- |
| RPH3AL | Body | chr17:113233-114319 | 8 | 2.08E-06 | 4.37E-05 | 0.392096 |
| HLA-DPB1 | Body | chr6:33048254-33049360 | 21 | 2.48E-05 | 0.000634 | 0.317255 |
| HLA-DRB5 | Body | chr6:32489801-32490444 | 4 | 0.000829 | 0.00143 | 0.287898 |
| WNT3 | Body | chr17:44847220-44847427 | 3 | 0.001472 | 0.001804 | 0.279832 |
| TMEM44 | Body | chr3:194342907-194343015 | 3 | 0.000939 | 0.001062 | 0.26746 |
| HS3ST3B1, MGC12916 | Body | chr17:14207241-14208363 | 7 | 2.20E-05 | 0.000507 | 0.264799 |
| GABBR1 | Body | chr6:29598695-29599319 | 9 | 0.002599 | 0.003852 | 0.259342 |
| CYP2E1 | Body | chr10:135341528-135342936 | 8 | 0.000357 | 0.000993 | 0.257594 |
| PRDM6 | Body | chr5:122433740-122435550 | 11 | 5.69E-08 | 5.95E-05 | 0.256947 |
| SPIB | Body, 3'UTR | chr19:50931222-50931875 | 7 | 0.000112 | 0.000411 | 0.249868 |
| HOXC4 | 5'UTR, TSS1500 | chr12:54446019-54446576 | 10 | 4.88E-05 | 8.08E-05 | 0.248858 |
| GNAS | 3'UTR, TSS1500,  Body, TSS200 | chr20:57463787-57464129 | 11 | 0.002489 | 0.003287 | 0.246738 |
| BARD1 | TSS1500 | chr2:215675429-215675637 | 3 | 6.75E-05 | 9.91E-05 | 0.243745 |
| CRB3 | TSS1500, TSS200,  5'UTR, 1stExon | chr19:6463949-6464563 | 11 | 0.003544 | 0.004503 | 0.236736 |
| HCG4P6 | TSS1500 | chr6:29894050-29894253 | 6 | 6.00E-05 | 6.58E-05 | 0.225232 |
| NIPAL2 | TSS200, TSS1500 | chr8:99306716-99306953 | 6 | 0.000973 | 0.001089 | 0.223072 |
| PXDNL | Body | chr8:52321814-52322341 | 7 | 0.00058 | 0.000739 | 0.218091 |
| FGFR2 | 5'UTR, 1stExon | chr10:123355576-123356041 | 3 | 0.00192 | 0.003073 | 0.216723 |
| SFN | TSS1500, TSS200,  1stExon, 5'UTR | chr1:27188505-27190330 | 12 | 9.68E-05 | 0.000409 | 0.213679 |
| PLAGL1 | TSS200, TSS1500 | chr6:144385792-144387124 | 21 | 6.81E-06 | 0.000613 | 0.212428 |
| ZNF469 | 1stExon, Body | chr16:88496241-88497293 | 6 | 1.50E-05 | 0.00075 | 0.206474 |
| FOXL1 | TSS1500, 1stExon,  5'UTR | chr16:86611049-86612451 | 5 | 1.62E-07 | 0.000397 | 0.202424 |
| TPPP3,  ZDHHC1 | TSS1500,3'UTR | chr16:67427811-67428324 | 4 | 0.000806 | 0.001623 | 0.200333 |
| CYP2C18 | TSS1500 | chr10:96442031-96442674 | 3 | 0.000243 | 0.000684 | -0.20398 |
| SHANK2 | Body | chr11:70564116-70564414 | 3 | 0.000335 | 0.001043 | -0.20487 |
| KRT5 | 1stExon,5'UTR,  TSS200,TSS1500 | chr12:52913824-52915064 | 7 | 3.11E-17 | 4.05E-07 | -0.20584 |
| PLEKHA4,  HSD17B14 | 3'UTR,TSS1500,  Body | chr19:49340489-49340765 | 4 | 0.000218 | 0.000268 | -0.20797 |
| LOC  100133612 | Body | chr1:3826803-3827428 | 4 | 0.001499 | 0.003142 | -0.21233 |
| CALCA | TSS1500 | chr11:14994381-14995770 | 25 | 1.05E-06 | 0.000305 | -0.21341 |
| MIR1228,  LRP1 | TSS1500,Body,  TSS200 | chr12:57587701-57588350 | 11 | 0.000688 | 0.001006 | -0.2181 |
| LHX3 | Body | chr9:139092321-139094665 | 10 | 3.34E-08 | 0.00027 | -0.23265 |
| KCNE1 | 5'UTR,1stExon,  TSS200,TSS1500 | chr21:35831871-35832364 | 10 | 0.000364 | 0.000428 | -0.26429 |
| DPP6 | Body,3'UTR | chr7:154684051-154685388 | 11 | 0.000769 | 0.002026 | -0.27278 |
| ISL2 | Body,3'UTR | chr15:76633086-76635953 | 14 | 8.16E-06 | 0.000401 | -0.27913 |
| HLA-DRB6 | Body | chr6:32525979-32526342 | 5 | 3.90E-08 | 2.72E-07 | -0.52578 |

**Table S1**: Summary of the 35 gene annotated regions containing a minimum of 3 CpG probes, and a maximum difference in DNA methylation of at least 20% (difference in beta value of 0.2). A positive delta beta identifies decreased DNA methylation in cells isolated from individuals with COPD, while a negative value identifies increased DNA methylation in cells isolated from individuals with COPD.
