# Supplementary material for: Altered DNA methylation is associated with aberrant gene expression in parenchymal but not airway fibroblasts isolated from individuals with COPD
Source: Clin Epigenetics. 2018 Mar 5;10:32. doi: 10.1186/s13148-018-0464-5 (PMC5838860; doi:10.1186/s13148-018-0464-5)
Supplement: Supplementary file 2 — Table S2. (.MWD): DNA regions differentially methylated with COPD status in parenchymal fibroblasts. Summary of the three gene annotated regions containing a minimum of three CpG probes and a maximum difference in DNA methylation of at least 20% (difference in beta value of 0.2). A positive delta beta identifies decreased DNA methylation in cells isolated from individuals with COPD, while a negative value identifies increased DNA methylation in cells isolated from individuals with COPD. (DOCX 14 kb) [file 13148_2018_464_MOESM2_ESM.docx]

| **Gene Associated** | **Gene region location** | **hg19 coordinate** | **Number of probes** | **Minimum p value** | **Mean p value** | **Maximum beta value difference** |
| --- | --- | --- | --- | --- | --- | --- |
| **HLX** | Body | chr1:221055097-221055964 | 7 | 4.50E-06 | 0.001011 | -0.21409 |
| **LOC100130872,**  **SPON2** | Body, TSS200,  TSS1500 | chr4:1201881-1203653 | 18 | 4.51E-07 | 0.000352 | -0.20321 |
| **NXN** | Body | chr17:805469-805576 | 3 | 0.000129 | 0.000133 | 0.261236 |

**Table S2**: Summary of the 3 gene annotated regions containing a minimum of 3 CpG probes, and a maximum difference in DNA methylation of at least 20% (difference in beta value of 0.2). A positive delta beta identifies decreased DNA methylation in cells isolated from individuals with COPD, while a negative value identifies increased DNA methylation in cells isolated from individuals with COPD.
